# Suppression of FOXM1 Sensitizes Human Cancer Cells to Cell Death Induced by DNA-Damage

**DOI:** 10.1371/journal.pone.0031761

**Published:** 2012-02-29

**Authors:** Marianna Halasi, Andrei L. Gartel

**Affiliations:** 1 Department of Medicine, University of Illinois, Chicago, Illinois, United States of America; 2 Department of Biochemistry and Molecular Genetics, University of Illinois, Chicago, Illinois, United States of America; 3 Department of Microbiology and Immunology, University of Illinois, Chicago, Illinois, United States of America; Sun Yat-sen University Medical School, China

## Abstract

Irradiation and DNA-damaging chemotherapeutic agents are commonly used in anticancer treatments. Following DNA damage FOXM1 protein levels are often elevated. In this study, we sought to investigate the potential role of FOXM1 in programmed cell death induced by DNA-damage. Human cancer cells after FOXM1 suppression were subjected to doxorubicin or γ-irradiation treatment. Our findings indicate that FOXM1 downregulation by stable or transient knockdown using RNAi or by treatment with proteasome inhibitors that target FOXM1 strongly sensitized human cancer cells of different origin to DNA-damage-induced apoptosis. We showed that FOXM1 suppresses the activation of pro-apoptotic JNK and positively regulates anti-apoptotic Bcl-2, suggesting that JNK activation and Bcl-2 down-regulation could mediate sensitivity to DNA-damaging agent-induced apoptosis after targeting FOXM1. Since FOXM1 is widely expressed in human cancers, our data further support the fact that it is a valid target for combinatorial anticancer therapy.

## Introduction

γ-irradiation and DNA-damaging chemotherapeutic drugs play a key role in anticancer therapy due to their ability to induce DNA double-strand breaks leading to cancer cell death [1]. Since cancer cells often become resistant to DNA-damaging agents, it is important to determine the mechanisms of drug-resistance. Several studies have reported that in response to IR, etoposide, daunorubicin or doxorubicin treatment FOXM1 protein level increases in a dose-dependent manner [2]–[4]. FOXM1 is considered to be a master regulator of the cell cycle [5], [6] by controlling the expression of genes that are crucial for G1/S and G2/M progression [7]. FOXM1 is abundantly expressed in a wide range of human cancers [8]–[11], suggesting that targeting FOXM1 could be a therapeutic strategy against human malignancies [12], [13]. FOXM1 has been implicated in the DNA-damage response pathway, for example DNA repair genes, XRCC1 and BRCA2 were identified as direct transcriptional targets of FOXM1 [2]. In addition, the role of FOXM1 in response to DNA-damage has been investigated in the context of human cancer cells with wild type p53 [2], [11], [14], [15]. However, tumor suppressor p53 was found to be a negative regulator of FOXM1 and DNA-damage strongly upregulated the level of FOXM1 in the absence of p53 [3], [4]. Consequently, we hypothesized that DNA-damaging chemotherapeutic agents may not be as efficient in the absence of p53, as they stabilize FOXM1 protein level leading to protection against DNA-damage-induced apoptosis. Since FOXM1 is potentially an oncogenic transcription factor and it is also involved in invasion and angiogenesis [16]–[24], treatment of tumors where p53 is mutated or inactivated with DNA-damaging agents could be detrimental for patients. Our group reported previously that suppression of FOXM1 by thiazole antibiotics [25]–[28] and by proteasome inhibitors [29] correlates with the degree of apoptosis suggesting that FOXM1 may act as a potential inhibitor of apoptosis [25]–[27]. Moreover, it has been shown recently that breast cancer cells with elevated levels of FOXM1 became insensitive to Herceptin, paclitaxel [30] and cisplatin [11]. Therefore, it is evident that FOXM1 might inhibit apoptosis induced by various anticancer drugs.

c-Jun N-terminal kinases (JNKs; also known as stress-activated protein kinases, SAPKs) respond to diverse extracellular stimuli and environmental stresses [31]. The JNK signaling pathway regulates many cellular processes, such as proliferation, survival, apoptosis and differentiation [32]. FOXM1 has been shown to transcriptionally activate JNK1 to control cell cycle progression and invasion [18]. However, the outcome of JNK activation is determined by the duration of JNK signaling [33], [34]. Short-term JNK activation is linked to proliferation, while sustained activation of JNK usually leads to apoptosis [33], [34]. B-cell lymphoma 2 (Bcl-2) is an antiapoptotic member of the Bcl-2 family [35], [36]. Bcl-2 has a central role in the intrinsic apoptotic pathway acting as the guardian of the mitochondria by inhibiting Bax activation [35], [37]. Forced overexpression of Bcl-2 in several cultured cell lines conferred resistance to chemotherapeutic agents and irradiation [35].

In this study, we examined whether FOXM1 plays a role in cell death induced by DNA-damage in human tumor cell lines with mutant p53. We demonstrate that stable or transient knockdown of FOXM1 using RNAi increases the sensitivity of different human cancer cells to DNA-damaging agents including doxorubicin treatment and γ-irradiation. Moreover, we show that combination of proteasome inhibitors that inhibit FOXM1 with DNA-damaging agents induces very robust apoptosis. Our findings suggest that JNK activation and Bcl-2 downregulation may account for the increased cell death after suppression of FOXM1 in combination with DNA-damage.

## Materials and Methods

### Cell culture, chemical compounds and treatments

**Figure 1:**
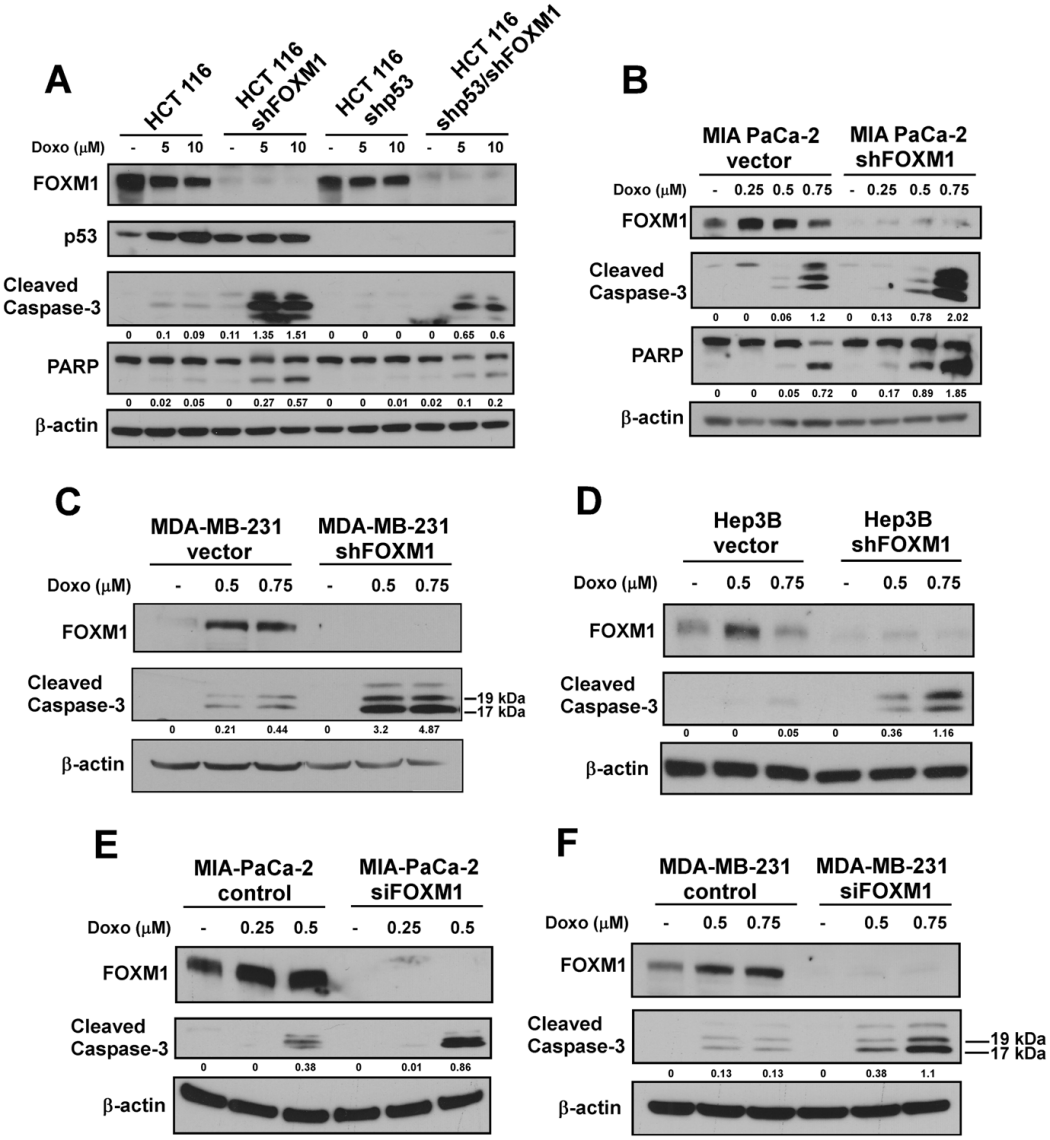
Suppression of FOXM1 by RNAi sensitizes human cancer cells to doxorubicin. (**A**) HCT 116 p53 proficient and deficient human colon cancer cells with FOXM1 knockdown were treated with doxorubicin (Doxo) as indicated. Immunoblotting for FOXM1, p53, cleaved caspase-3, PARP and β-actin as the loading control was carried out 24 hours after treatment. (**B**) MIA PaCa-2 vector control and FOXM1 knockdown pancreatic cancer cells were treated with the indicated concentrations of doxorubicin. Twenty-four hours after treatment cell lysates were immunoblotted for FOXM1, cleaved caspase-3, PARP and β-actin as the loading control. (**C**) MDA-MB-231 vector control and FOXM1 knockdown breast cancer cells were treated with doxorubicin as indicated. Immunoblot analysis was performed for FOXM1, cleaved caspase-3 and β-actin as the loading control 48 hours after treatment. (**D**) Hep3B vector control and FOXM1 knockdown liver cancer cells were treated with the indicated concentrations of doxorubicin. Cell lysates were immunoblotted for FOXM1, cleaved caspase-3 and β-actin as the loading control 24 hours after treatment. (**E**) Pancreatic cancer cells MIA PaCa-2 were transfected with control and FOXM1 siRNA for 48 hours, and then treated as indicated. Twenty-four hours after treatment immunoblotting was carried out with FOXM1, cleaved caspase-3 and β-actin antibodies. (**F**) Breast cancer cells MDA-MB-231 were transfected with control and FOXM1 siRNA for 48 hours, then treated with the indicated concentrations of doxorubicin. Forty-eight hours after treatment cell lysates were immunoblotted for FOXM1, cleaved caspase-3 and β-actin as the loading control.

MIA PaCa-2 pancreatic (ATCC), Hep3B liver (ATCC), HCT 116 and HCT 116 shp53 colon [38] human cancer cell lines were grown in DMEM medium (Invitrogen). MDA-MB-231 (ATCC) breast human cancer cell line was grown in RPMI medium (Invitrogen). The media were supplemented with 10% fetal bovine serum (Atlanta Biologicals) and 1% penicillin-streptomycin (GIBCO) and the cells were kept at 37°C in 5% CO_2_. Stable cell lines (Fig. 1) using the ATCC obtained parental cells were generated by transduction of control and FOXM1 shRNA lentiviral particles (Sigma) followed by selection with puromycin (Sigma). Doxorubicin (Fisher Scientific), thiostrepton (Sigma), bortezomib (velcade) (Millenium Pharmaceuticals) and JNK inhibitor SP600125 (A.G. Scientific) were dissolved in dimethyl sulfoxide (Fisher Scientific). Irradiation was carried out using a ^137^Caesium γ-source (J. L. Shepherd model 6810; J. L. Shepherd, San Fernando, CA) at a dose of 8 and 10 Gy.

### Immunoblot analysis

Treated cells were harvested and processed for immunoblotting as described in ref. [27] with antibodies specific for FOXM1 (the rabbit polyclonal antibody against FOXM1 was described previously [45]), p53 (Santa Cruz), cleaved caspase-3 (Cell signaling), PARP-1/2 (Santa Cruz), Total and Phospho-SAPK/JNK (Cell signaling), Bcl-2 (Santa Cruz), Bcl-xL (Cell signaling) and β-actin (Sigma). Quantification was done with the Image J software (NIH). Relative protein levels were normalized to the corresponding actin levels.

### Transfection and siRNA

Control (AACAGUCGCGUUUGCGACUGGUU) small interfering RNA (siRNA) and siRNA specific to FOXM1 (GGACCACUUUCCCUACUUUUU) were synthesized by Sigma. 50 nM of siRNA duplexes were transfected into cells using Lipofectamine 2000 (Invitrogen) according to the manufacturer's recommendation. Cells were treated as indicated 48 hours after transfection.

### Total RNA extraction and Quantitative real-time (qRT)-PCR

To extract total RNA cells were harvested by TRIzol reagent (Invitrogen). cDNA was synthesized using the High Capacity cDNA Reverse Transcription Kit (Applied Biosystems). Quantitative real time PCR was run using the ABI 7900 HT (Applied Biosystems) machine. The following primers were used: human Bcl-2 (Sense, 5′-TGG GAT GCC TTT GTG GAA CT-3′; Antisense, 5′-GAG ACA GCC AGG AGA AAT CAA AC-3′) [46] and human cyclophilin (Sense, 5′-GCA GAC AAG GTC CCA AAG ACA G-3′; Antisense, 5′-CAC CCT GAC ACA TAA ACC CTG G-3′) [25].

### Flow cytometry – Propidium Iodide staining

Cells were treated as indicated and harvested by trypsinization. Then, cells were washed in PBS and fixed in ice-cold 95% ethanol. Following fixation, cells were stained with propidium iodide (50 µg/ml) (Invitrogen) in PBS/RNAse A/Triton X-100 for 30 min at room temperature and analyzed by flow cytometry.

### Colony-forming assay

1×10^5^ cells were plated on 100 mm dishes in duplicates and were treated as indicated for 24 hrs. Colonies were allowed to form for 10 days, and then cells were stained with crystal violet.

### Statistical analysis

Statistical analysis was performed with Microsoft Excel using the Student *t* test (two-tailed). *P* values of <0.05 were considered to be statistically significant.

## Results and Discussion

### Knockdown of FOXM1 sensitizes human cancer cells to DNA-damaging agents

First, isogenic HCT 116 p53 proficient and deficient human colon cancer cells with FOXM1 knockdown were treated with different concentrations of doxorubicin (Doxo) and cell death was assessed by immunoblotting for markers of apoptosis including cleaved caspase-3 and PARP (Fig. 1A). Knockdown of FOXM1 enhanced sensitivity of colon cancer cells to doxorubicin-mediated apoptosis independently of p53 status. p53 proficient cells with FOXM1 knockdown underwent stronger apoptosis compared to their p53 deficient counterparts. This observation could be explained by the presence of p53-dependent apoptotic signaling in the p53-proficient cells, but not in the p53-deficient cells following DNA-damage.

About 50% of human tumors harbor mutant or inactivated p53; consequently the p53-dependent apoptotic pathway cannot be exploited for the eradication of cancer cells following anticancer treatment [39]. To investigate the possible role of FOXM1 in DNA-damage-induced apoptosis in cell lines with mutant p53, which are usually more resistant to chemotherapy than cancer cell lines with wild-type p53, MIA PaCa-2 vector control and FOXM1 knockdown human pancreatic cancer cell lines harboring mutant p53 were treated with increasing concentrations of doxorubicin and immunoblotting was performed for FOXM1, cleaved caspase-3 and PARP (Fig. 1B). In accordance with earlier studies [2]–[4], [11], [14], [40], we also observed that following DNA-damage FOXM1 protein level is elevated. But more importantly, we found that the absence of FOXM1 sensitized cancer cells to cell death induced by doxorubicin (Fig. 1B) as detected by the cleavage of caspase-3 and PARP. To verify that this phenomenon is not cell line specific, MDA-MB-231 human breast (p53-mutant) (Fig. 1C) and Hep3B human liver (p53-null) (Fig. 1D) vector control and FOXM1 knockdown cancer cells were treated with different concentrations of doxorubicin. Western blot analysis of cleaved caspase-3 demonstrated that knockdown of FOXM1 also sensitized breast and liver cancer cells to doxorubicin-induced apoptosis.

Additionally, we tested whether transient knockdown of FOXM1 results in increased apoptosis following DNA-damage. To attain sufficient transient knockdown of FOXM1 we utilized small interfering RNAs (siRNAs) against FOXM1. MIA PaCa-2 pancreatic (Fig. 1E) and MDA-MB-231 breast (Fig. 1F) control and FOXM1 knockdown cancer cells were treated with various concentrations of doxorubicin. We found that cells with transient FOXM1 knockdown were more susceptible to apoptosis after DNA-damage as detected by immunoblotting for cleaved caspase-3, further supporting the idea that FOXM1 might play a role in DNA-damage-induced apoptosis.

**Figure 2:**
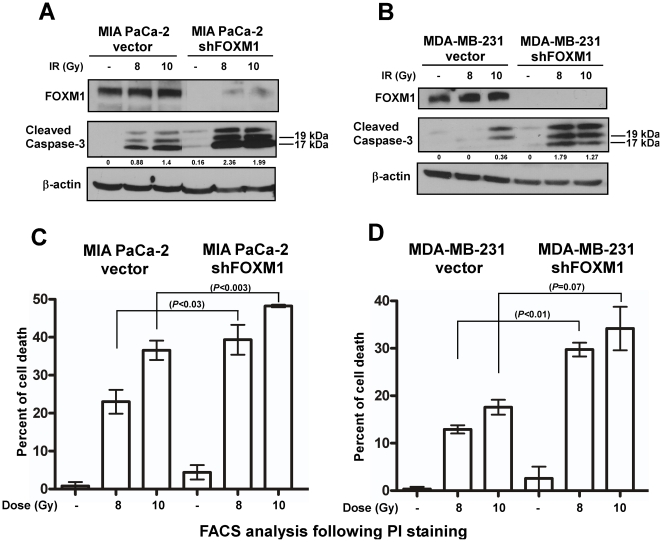
Suppression of FOXM1 by RNAi sensitizes human cancer cells to irradiation. (**A**) MIA PaCa-2 control and FOXM1 knockdown pancreatic cancer cells were γ-irradiated with 8 and 10 Gy. Seventy-two hours after irradiation cells were harvested and immunoblotting was carried out with antibodies for FOXM1 and cleaved caspase-3. β-actin was used as the loading control. (**B**) MDA-MB-231 control and FOXM1 knockdown breast cancer cells were subjected to γ-irradiation with the indicated doses. Forty-eight hours following irradiation cells were harvested and immunoblotting was performed for FOXM1, cleaved caspase-3 and β-actin as the loading control. (**C–D**) Degree of cell death was assessed by flow cytometry following propidium iodide staining in MIA PaCa-2 pancreatic and MDA-MB-231 breast control and FOXM1 knockdown cancer cells, respectively, 48 hours after γ-irradiation. Graph shows quantification as a percentage of apoptotic cells compared to the vector control nontreated cells, ±SD of a representative triplicate (**C**) or duplicate (**D**) experiment.

In order to confirm this effect, MIA PaCa-2 and MDA-MB-231 vector control and FOXM1 knockdown cells were subjected to ionizing radiation. γ-irradiation also induced robust apoptosis as detected by the cleavage of caspase-3 in FOXM1 knockdown cells (Fig. 2A, B). In addition, to quantitatively assess the degree of cell death induced by γ-irradiation MIA PaCa-2 and MDA-MB-231 vector control and FOXM1 knockdown cells were γ-irradiated with different doses, harvested and stained with propidium iodide. The extent of apoptosis was measured by flow cytometry (Fig. 2C, D). Both pancreatic and breast FOXM1 knockdown cells exhibited significant increase in cell death following treatment compared to the irradiated control counterparts. Altogether, these data suggest that suppressing FOXM1 in human cancer cells could make them more susceptible to cell death induced by DNA-damaging agents.

### Treatment with proteasome inhibitors sensitizes human cancer cells to apoptosis induced by DNA-damage

**Figure 3:**
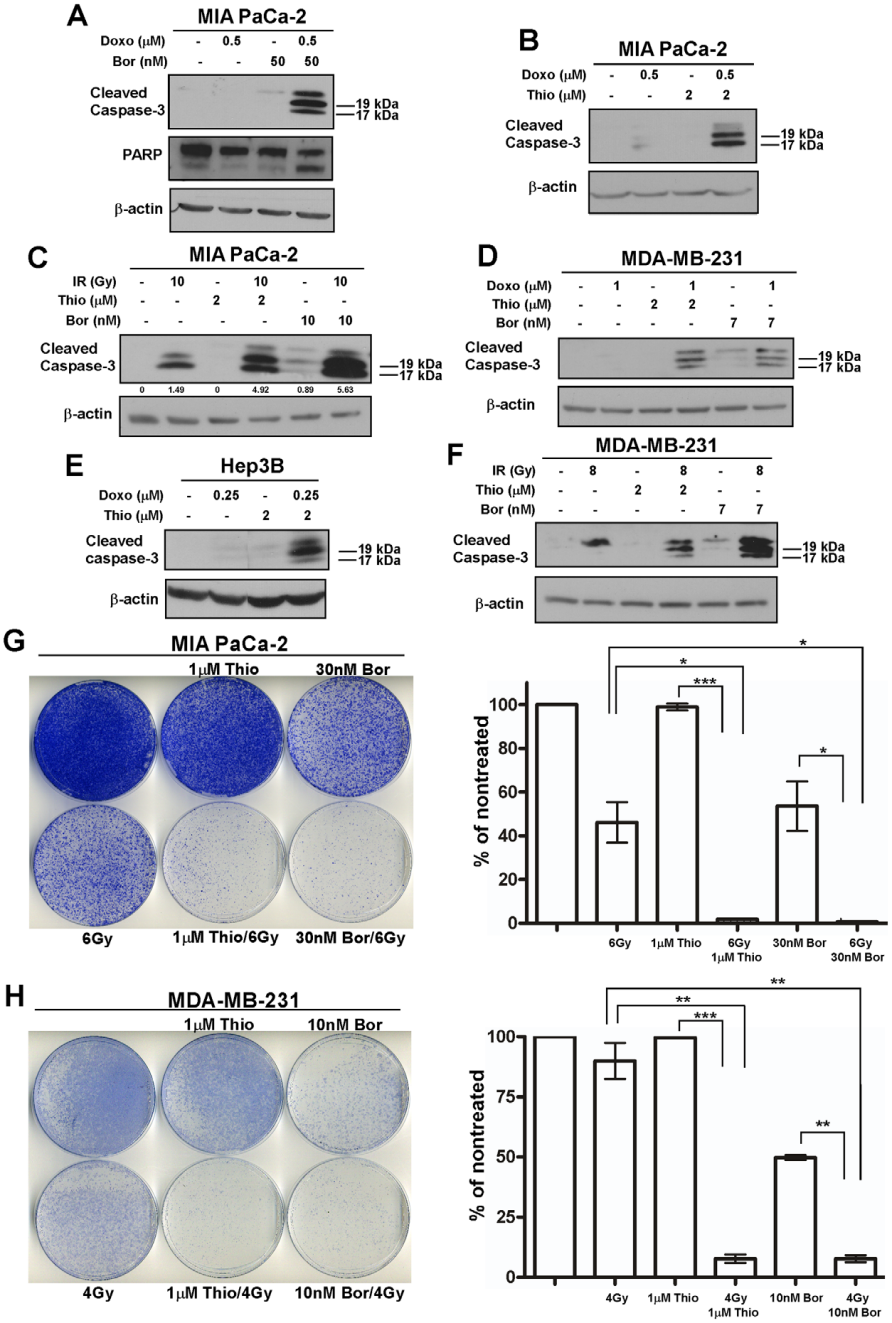
Combination of FOXM1/Proteasome inhibitors and DNA-damaging agents sensitizes human cancer cells to programmed cell death. (**A**) Pancreatic cancer cells MIA PaCa-2 were treated with the indicated concentrations of doxorubicin and bortezomib (Bor) alone or in combination. 24 hours after treatment cell lysates were analyzed by immunoblotting with cleaved caspase-3, PARP and β-actin antibodies. (**B**) MIA PaCa-2 pancreatic cancer cells were treated with doxorubicin and thiostrepton (Thio) alone or in combination with the indicated concentrations for 24 hours. Immunoblotting was performed with antibodies specific for cleaved caspase-3 and β-actin as the loading control. (**C**) Pancreatic cancer cell line MIA PaCa-2 was exposed to γ-irradiation and treated in combination with thiostrepton or bortezomib for 24 hours. Immunoblotting was carried out for cleaved caspase-3 and β-actin as the loading control. (**D**) MDA-MB-231 breast cancer cells were treated with doxorubicin in combination with thiostrepton or bortezomib as indicated for 24 hours. Cell lysates were analyzed by immunoblotting with antibodies specific for cleaved caspase-3 and β-actin as the loading control. (**E**) Hep3B liver cancer cells were treated with doxorubicin and thiostrepton alone or in combination as indicated for 24 hours. Immunoblotting was performed with antibodies specific for cleaved caspase-3 and β-actin as the loading control. (**F**) Breast cancer cell line MDA-MB-231 was subjected to γ-irradiation and treated in combination with thiostrepton or bortezomib for 24 hours. Immunoblot analysis was performed with cleaved caspase-3 and β-actin antibodies. (**G–H**) 1×10^5^ MIA PaCa-2 (G) or MDA-MB-231 (H) human cancer cells were plated and treated as indicated for 24 hours. 10 days after treatment cells were stained with crystal violet and representative plates are shown. Graph shows the quantification ±SD of duplicate experiments (*, *P*<0.05; **, *P*<0.01; ***, *P*<0.001).

Our group has reported previously that thiazole antibiotics thiostrepton and Siomycin A are potent inhibitors of FOXM1 [25]–[28] and act as proteasome inhibitors [29]. Furthermore, we have demonstrated that well-known proteasome inhibitors such as bortezomib (velcade) and MG132 also target FOXM1 [29]. Downregulation of FOXM1 is one of the means, by which proteasome inhibitors may induce cell death in vitro and in vivo [27], [29], [41], but there are of course several potential FOXM1-independent mechanisms of proteasome inhibitor-induced cell death that we are not discussing in this study. To test FOXM1/proteasome inhibitors together with DNA-damaging agents, MIA PaCa-2 pancreatic cancer cell line was treated with the combination of doxorubicin and bortezomib (Bor) (Fig. 3A) or thiostrepton (Thio) (Fig. 3B), respectively. We found that inhibitors of FOXM1, thiostrepton and bortezomib suppressed FOXM1 expression (data not shown) [27], [29] and in combination with doxorubicin exhibited stronger cleavage of caspase-3 or PARP compared to treatments with drugs as single agents. In addition, MIA PaCa-2 pancreatic cancer cells were γ-irradiated along with the treatment of thiostrepton or bortezomib (Fig. 3C). Western blot analysis for cleaved caspase-3 demonstrated that MIA PaCa-2 cells were more sensitive to the combination of γ-irradiation and thiostrepton or bortezomib, respectively, than to individual drug treatment.

To further verify this effect, MDA-MB-231 breast and Hep3B liver cancer cells were treated with doxorubicin (Fig. 3D, E) or irradiated (Fig. 3F) in combination with thiostrepton or bortezomib, respectively. Analysis of cleaved caspase-3 expression by western blot revealed that the combination of DNA-damaging agents with FOXM1/proteasome inhibitors led to increased apoptosis in breast and liver cancer cells compared to treatment with drugs alone. Since treatment with DNA-damaging agents in conjunction with FOXM1/proteasome inhibitors induced such marked apoptosis, the effect of their combination was also examined on long-term survival by performing clonogenic assay. MIA PaCa-2 and MDA-MB-231 human cancer cells were treated with γ-irradiation along with thiostrepton or bortezomib and the colony forming ability was assessed 10 days later (Fig. 3G, H). We found that the combination of γ-irradiation and FOXM1/proteasome inhibitors significantly inhibited colony formation giving rise to much less number of colonies compared to individual drug treatment. Taken together, these data suggest that the combination of commonly used DNA-damaging chemotherapeutic agents and FOXM1/proteasome inhibitors could be considered as a treatment strategy in order to improve therapeutic response in various cancer treatments.

### Sensitivity to DNA-damage after suppression of FOXM1 is partially attributed to JNK activation and Bcl-2 downregulation

**Figure 4:**
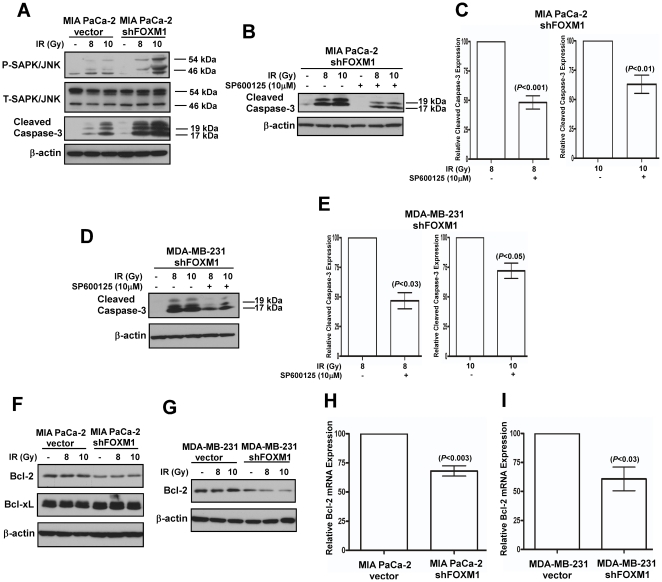
FOXM1 modulates JNK activity and Bcl-2 expression to sensitize human cancer cells to DNA-damage. (**A**) MIA PaCa-2 control and FOXM1 knockdown pancreatic cancer cells were γ-irradiated with the indicated doses. Seventy-two hours following irradiation cells were harvested and immunoblotting was carried out with antibodies specific for Phospho/Total-SAPK/JNK and cleaved caspase-3. β-actin was used as the loading control. (**B**) MIA PaCa-2 FOXM1 knockdown pancreatic cancer cells were preincubated for 1 hour with 10 µM of JNK inhibitor, SP600125 and then were γ-irradiated as indicated. Forty-eight hours following irradiation cells were harvested and immunoblotting was performed for cleaved caspase-3 and β-actin as the loading control. (**C**) The graphs show mean values ± SEM of four independent experiments. (**D**) MDA-MB-231 FOXM1 knockdown breast cancer cells were preincubated for 1 hour with 10 µM of JNK inhibitor, SP600125 and then were subjected to γ-irradiation with the indicated doses. Seventy-two hours after irradiation cells were harvested and immunoblotting was performed with cleaved caspase-3 and β-actin antibodies. (**E**) The graphs show mean values ± SEM of two independent experiments. (**F**) Pancreatic MIA PaCa-2 control and FOXM1 knockdown cancer cells were subjected to ionizing radiation with the indicated doses. Thirty-six hours following irradiation cells were harvested and immunoblotting was carried out with antibodies specific for Bcl-2 and Bcl-xL. β-actin was used as the loading control. (**G**) Breast MDA-MB-231 control and FOXM1 knockdown cancer cells were γ-irradiated as indicated. Seventy-two hours following irradiation cells were harvested and immunoblotting was performed for Bcl-2. β-actin was used as the loading control. (**H**) MIA PaCa-2 control and FOXM1 knockdown pancreatic cancer cells were harvested for RNA extraction. Quantitative RT-PCR was carried out with Bcl-2 and cyclophilin primers. The graph demonstrates mean values ±SEM of three independent experiments. (**I**) To extract RNA MDA-MB-231 control and FOXM1 knockdown breast cancer cells were harvested. Using Bcl-2 and cyclophlin primers qRT-PCR was performed. The graph shows mean values ±SEM of three independent experiments.

It is an intriguing fact that FOXM1, a known regulator of the cell cycle, may inhibit DNA-damage-induced apoptosis. In order to elucidate the possible underlying mechanism we turned to the JNK signaling pathway, because it has been implicated in responding to diverse stimuli including various apoptotic stimuli [32]. It has been reported that γ-irradiation leads to JNK activation, which mediates radiation-induced apoptosis [33], [42]–[44]. To determine the effect of DNA-damage on the activation of JNK in the presence or absence of FOXM1, MIA PaCa-2 vector control and FOXM1 knockdown pancreatic cancer cells were γ-irradiated and immunoblotted for phospho-SAPK/JNK (Fig. 4A). We observed that in FOXM1-knockdown cells the induction of cell death following γ-irradiation was accompanied by elevated phosphorylation of JNK, suggesting that FOXM1 may inhibit the activation of JNK in the presence of DNA-damage. In order to investigate the potential role of JNK activation in apoptosis induced by DNA-damage in FOXM1 knockdown cells, MIA PaCa-2 pancreatic and MDA-MB-231 breast FOXM1 knockdown cancer cells were γ-irradiated in the presence of the specific JNK inhibitor, SP600125 (Fig. 4B–E). Western blot analysis for cleaved caspase-3 showed that induction of apoptosis by irradiation was attenuated by treatment with the JNK inhibitor, suggesting that JNK activation is partially responsible for the programmed cell death following DNA-damage.

We also looked for additional mechanisms that might be involved in the sensitization to DNA-damage in the absence of FOXM1. The Bcl-2 family of proteins is one of the major regulators of apoptosis [35]–[37]. Bcl-2 is best known to prevent apoptosis by governing mitochondrial membrane integrity [35]–[37]. Bcl-2 was also found to confer resistance to chemotherapeutic agents and irradiation in various cell lines [35]. Immunoblot analysis of Bcl-2 and Bcl-xL expression after γ-irradiation revealed that Bcl-2 was downregulated in FOXM1 knockdown pancreatic and breast cancer cells (Fig. 4F, G), while Bcl-xL protein level remained unchanged (Fig. 4F). To examine whether Bcl-2 is a transcriptional target of FOXM1, Bcl-2 mRNA expression was assessed by quantitative real-time (qRT)-PCR in vector control and FOXM1 knockdown pancreatic and breast cancer cells. FOXM1 knockdown led to 30–40% reduction in Bcl-2 mRNA expression (Fig. 4H, I), suggesting that FOXM1 transcriptionally up-regulates Bcl-2. Future experiments are needed to determine whether Bcl-2 is a direct target of FOXM1. These data suggest that suppression of FOXM1 might also sensitize human cancer cells to DNA-damage via Bcl-2 downregulation.

In summary, we demonstrated that FOXM1 suppression by stable or transient knockdown using RNAi (Fig. 1, 2) or by treatment with FOXM1/proteasome inhibitors (Fig. 3) sensitizes human cancer cells of different origin to apoptosis induced by DNA-damaging agents including doxorubicin and γ-irradiation. The effect of endogenous FOXM1 knockdown in human cancer cells on apoptosis after doxorubicin and γ-irradiation treatment has never been investigated before. JNK activation and Bcl-2 downregulation as a result of FOXM1 knockdown may explain the resistance of FOXM1 proficient cells to apoptosis following DNA-damage. Overall, our findings in accordance with previous reports [11], [14] confirm that targeting FOXM1 in combination with DNA-damaging drugs or with γ-irradiation could be a valuable therapeutic approach against different types of human cancer. Since some of the FOXM1/proteasome inhibitors such as bortezomib are already in clinical practice, the data presented here also suggest that the administration of commonly used DNA-damaging chemotherapeutic agents in conjunction with FOXM1/proteasome inhibitors or with other potential FOXM1 inhibitors should be warranted serious consideration in order to improve therapeutic outcome.
